# Effect of test duration and sensor location on the reliability of standing balance parameters derived using body-mounted accelerometers

**DOI:** 10.1186/s12938-023-01196-7

**Published:** 2024-01-02

**Authors:** Vahid Abdollah, Alireza Noamani, John Ralston, Chester Ho, Hossein Rouhani

**Affiliations:** 1 Department of Mechanical Engineering, University of Alberta, Edmonton, AB Canada; 2 Division of Physical Medicine and Rehabilitation, University of Alberta, Edmonton, AB Canada; 3 Neursantys Inc., Calgary, AB Canada; 4 Glenrose Rehabilitation Hospital, Edmonton, AB Canada

**Keywords:** Inertial wearable sensors, Standing balance, Test duration, IMU location, Accelerometer

## Abstract

**Background:**

Balance parameters derived from wearable sensor measurements during postural sway have been shown to be sensitive to experimental variables such as test duration, sensor number, and sensor location that influence the magnitude and frequency-related properties of measured center-of-mass (COM) and center-of-pressure (COP) excursions. In this study, we investigated the effects of test duration, the number of sensors, and sensor location on the reliability of standing balance parameters derived using body-mounted accelerometers.

**Methods:**

Twelve volunteers without any prior history of balance disorders were enrolled in the study. They were asked to perform two 2-min quiet standing tests with two different testing conditions (eyes open and eyes closed). Five inertial measurement units (IMUs) were employed to capture postural sway data from each participant. IMUs were attached to the participants’ right legs, the second sacral vertebra, sternum, and the left mastoid processes. Balance parameters of interest were calculated for the single head, sternum, and sacrum accelerometers, as well as, a three-sensor combination (leg, sacrum, and sternum). Accelerometer data were used to estimate COP-based and COM-based balance parameters during quiet standing. To examine the effect of test duration and sensor location, each 120-s recording from different sensor locations was segmented into 20-, 30-, 40-, 50-, 60-, 70-, 80-, 90-, 100-, and 110-s intervals. For each of these time intervals, time- and frequency-domain balance parameters were calculated for all sensor locations.

**Results:**

Most COM-based and COP-based balance parameters could be derived reliably for clinical applications (Intraclass-Correlation Coefficient, ICC ≥ 0.90) with a minimum test duration of 70 and 110 s, respectively. The exceptions were COP-based parameters obtained using a sacrum-mounted sensor, especially in the eyes-closed condition, which could not be reliably used for clinical applications even with a 120-s test duration.

**Conclusions:**

Most standing balance parameters can be reliably measured using a single head- or sternum-mounted sensor within a 120-s test duration. For other sensor locations, the minimum test duration may be longer and may depend on the specific test conditions.

**Supplementary Information:**

The online version contains supplementary material available at 10.1186/s12938-023-01196-7.

## Background

Balance deficits are an important component of most neurological and many musculoskeletal disorders [1]. Balance deficits are usually evaluated using qualitative parameters or semi-quantitative scoring tools. Standardized clinical scales such as Berg Balance, Romberg’s, or Pull test are commonly administered in clinical settings to identify balance deficits. Although they can be administrated quickly, the limited accuracy and intrinsic subjectivity of these tests significantly reduce their reliability [2]. Furthermore, they suffer from low diagnostic sensitivity and specificity, low power to predict the risk of falling, and limited utility for the assessment of disabilities in the performance of daily activities [3, 4]. Other limitations of standardized clinical scales are their nonlinear distribution and floor or ceiling effects, particularly when individuals with subtle balance deficits are evaluated [5].

Inherent limitations of clinical scales have driven efforts to deploy laboratory instruments such as force-plates, computerized dynamic posturography systems, and optoelectronic cameras to improve the objectivity of the balance assessments. Standing balance has been evaluated based on both body center-of-pressure (COP) and center-of-mass (COM) [6–8]. Despite the close relationship between the COP and COM, especially when averaged over time, instantaneous COM and COP likely reflect distinct aspects of movement and neural control [9]. Muscular activity for maintaining balance is likely coordinated and activated in response to COM movements [9]. Thus, the stability of COM movement is a performance indicator for the neuromuscular control system. At the same time, COP movements are closely related to the ankle joint moment and provide information on the neuromuscular control system’s efforts to maintain COM stability and standing balance [9, 10]. In a one-segment inverted pendulum model of the body, the COP trajectory is obtained as a function of both the COM trajectory and COM acceleration [11]. When a multi-segment model of the body is employed, the relationship between COP and COM becomes more complex [12]. Therefore, COP and COM sway provide different information about standing balance, and both should be evaluated for clinical or research purposes.

Despite the large volume of research conducted using motion-capture systems (for COM tracking) and force-plates (for COP tracking), their broader adoption for clinical practice is limited by a (i) their high cost and requirement for dedicated technical expertise, which reduces their utility outside laboratory environments [13, 14] and (ii) their incompatibility with continuous or remote monitoring, the importance and value of which were highlighted by the COVID-19 pandemic [15, 16]. Wearable devices are especially attractive for remote health monitoring, clinical outcome evaluation, and earlier detection of disease progression and trajectories of long-term outcomes [17–19]. Wearable inertial measurement units (IMUs) have shown great promise for characterizing balance in the clinic and the community and have achieved levels of accuracy that make them a highly effective tool for clinical purposes [8, 12, 15, 20, 21]. They can be used to objectively evaluate individuals’ balance with minimum preparation time or technical expertise. Moreover, they enable clinicians to monitor neurological symptoms continuously and examine patient trends over time [22].

Recently, we proposed and validated a wearable system based on accelerometers to concurrently obtain the COM and COP trajectories during standing [12]. Due to the non-stationary characteristics of COM and COP excursions [23–25], the magnitude- and frequency-related properties of measured COM and COP excursions and balance parameters derived from COP and COM may vary as a function of test duration. Different test durations (between 20 and 364 s) have been used to evaluate postural control [26–33]. A 30-s test duration is the most common test duration reported in balance studies for accelerometers mounted on the lower back [27–31]. However, further studies are needed to provide experimental support for choosing test duration when balance parameters are measured using IMUs [34].

There is also a lack of consistency in the literature regarding the location and the number of wearable sensors for balance assessments. We previously showed that combining data from multiple sensors can improve the accuracy of balance measurements by supporting analysis via multi-segment models of the body [12]. These multi-segment models are able to replicate more complex body motions, which in turn deliver more accurate estimations of COM and COP trajectories. However, the use of multiple sensors can also increase the complexity and obtrusiveness of the test apparatus, which is why many researchers still prefer to use a single sensor [8, 12, 21, 35]. Mancini et al. [8] employed a waist-mounted accelerometer to estimate seven time-domain and five frequency-domain COM acceleration balance parameters originally proposed by Prieto et al. [7]. They used a test duration of 30 s for data collection [8]. Ralston et al. employed an IMU mounted on the mastoid process to estimate new “Phybrata” balance parameters [35] and concluded that 20 s is sufficient for the derivation of these new parameters [35]. Reynard et al. employed a chest-mounted accelerometer to assess the utility of a battery of quiet standing tasks for the assessment of postural control [14]. They used a test duration of 30 s for data collection [14].

Inconsistent selection of sensor location, number of sensors, and test duration in the literature may account for inconsistent conclusions and, consequently, inconsistent integration of wearable sensors into conventional balance assessment methodologies for research or clinical applications. Thus, there is a need to investigate the effect of these factors on measurement outcomes [36]. The primary objective of this study was, therefore, to investigate the effects of test duration, the number of sensors, and sensor location on the reliability of balance parameters obtained using IMU measurements. We hypothesized that (i) the reliability of computed balance parameters will change as a function of the test duration, but these changes will diminish beyond a ‘required minimum test duration’, and (ii) this ‘required minimum test duration’ will vary with the sensor location, the number of sensors, and testing conditions (such as eyes open vs. eyes closed).

## Results

All participants completed the study.

### Change in balance parameters

**Fig. 1:**
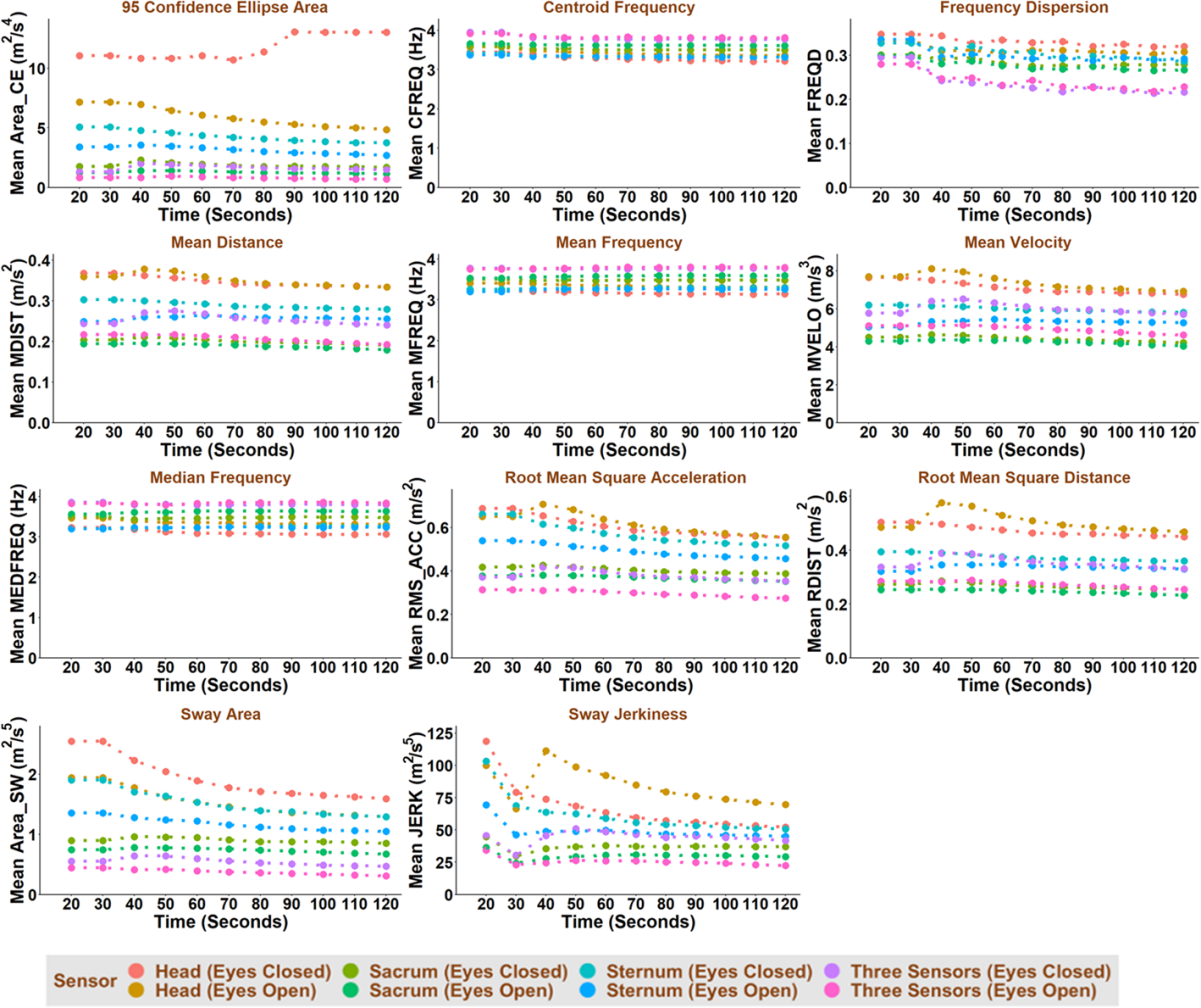
COM-based balance parameters as a function of test duration, test condition and sensor location for anteroposterior direction

**Fig. 2:**
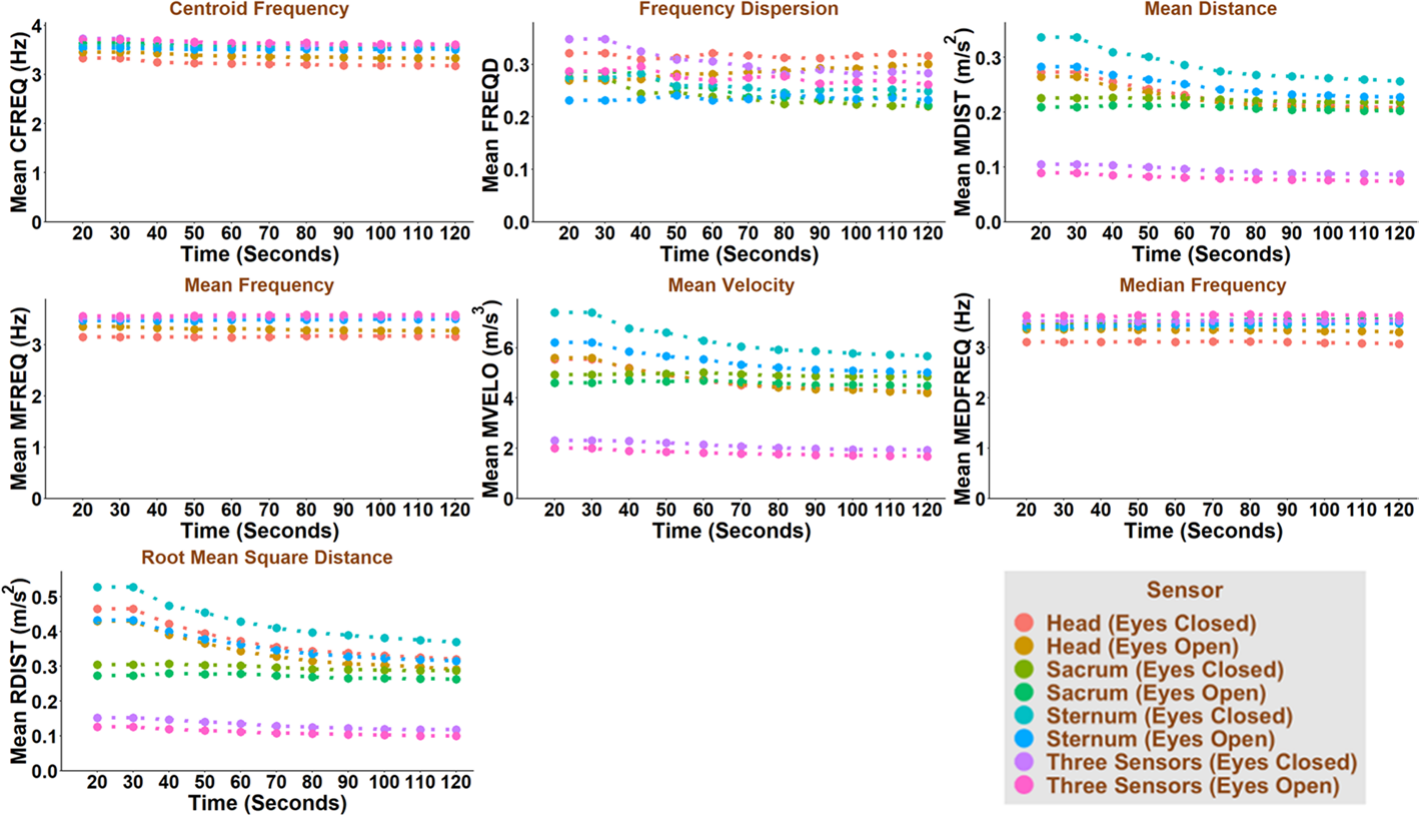
COM-based balance parameters as a function of test duration, test condition and sensor location for mediolateral direction

**Fig. 3:**
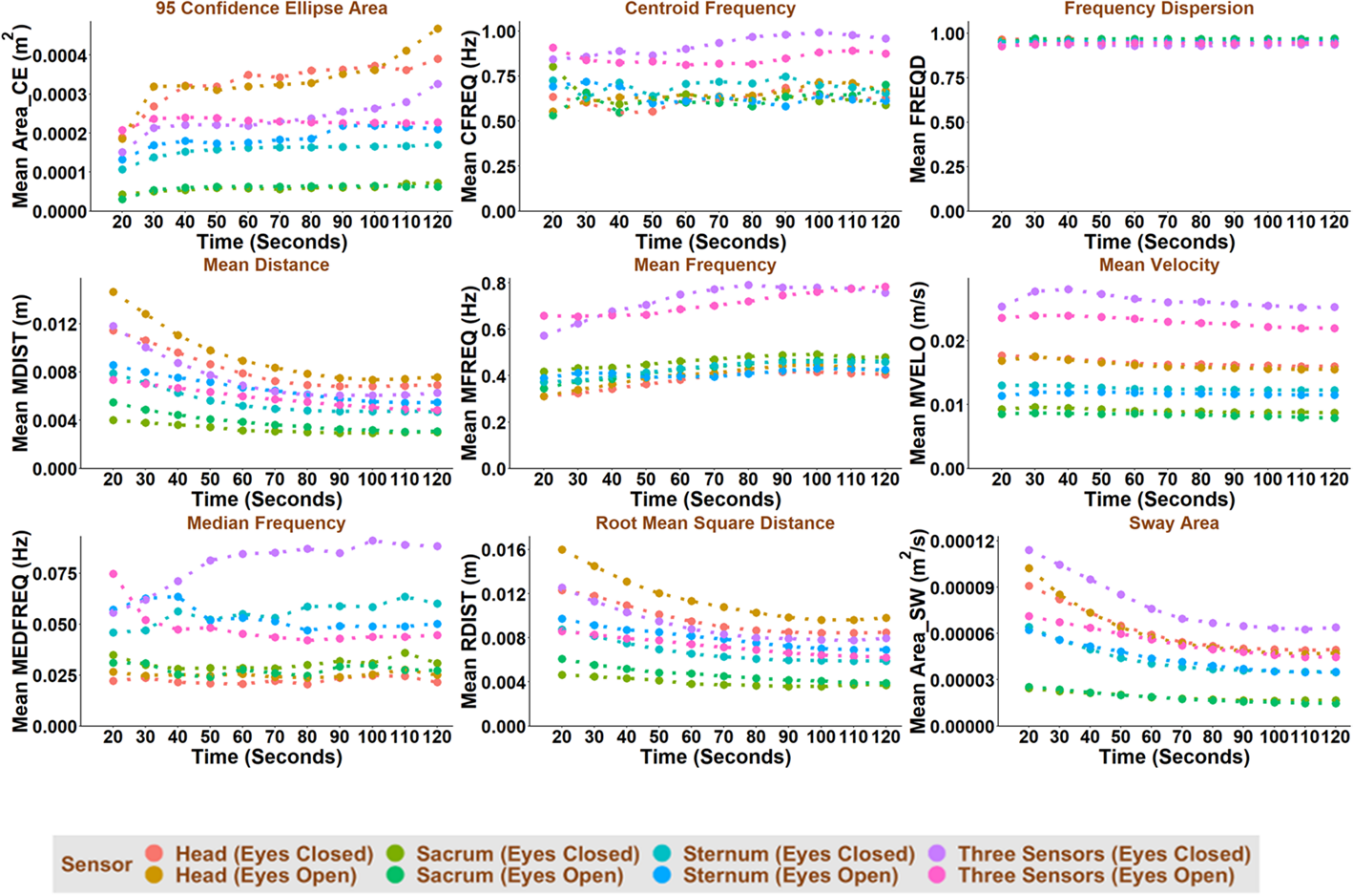
COP-based balance parameters as a function of test duration, test condition and sensor location for anteroposterior direction

**Fig. 4:**
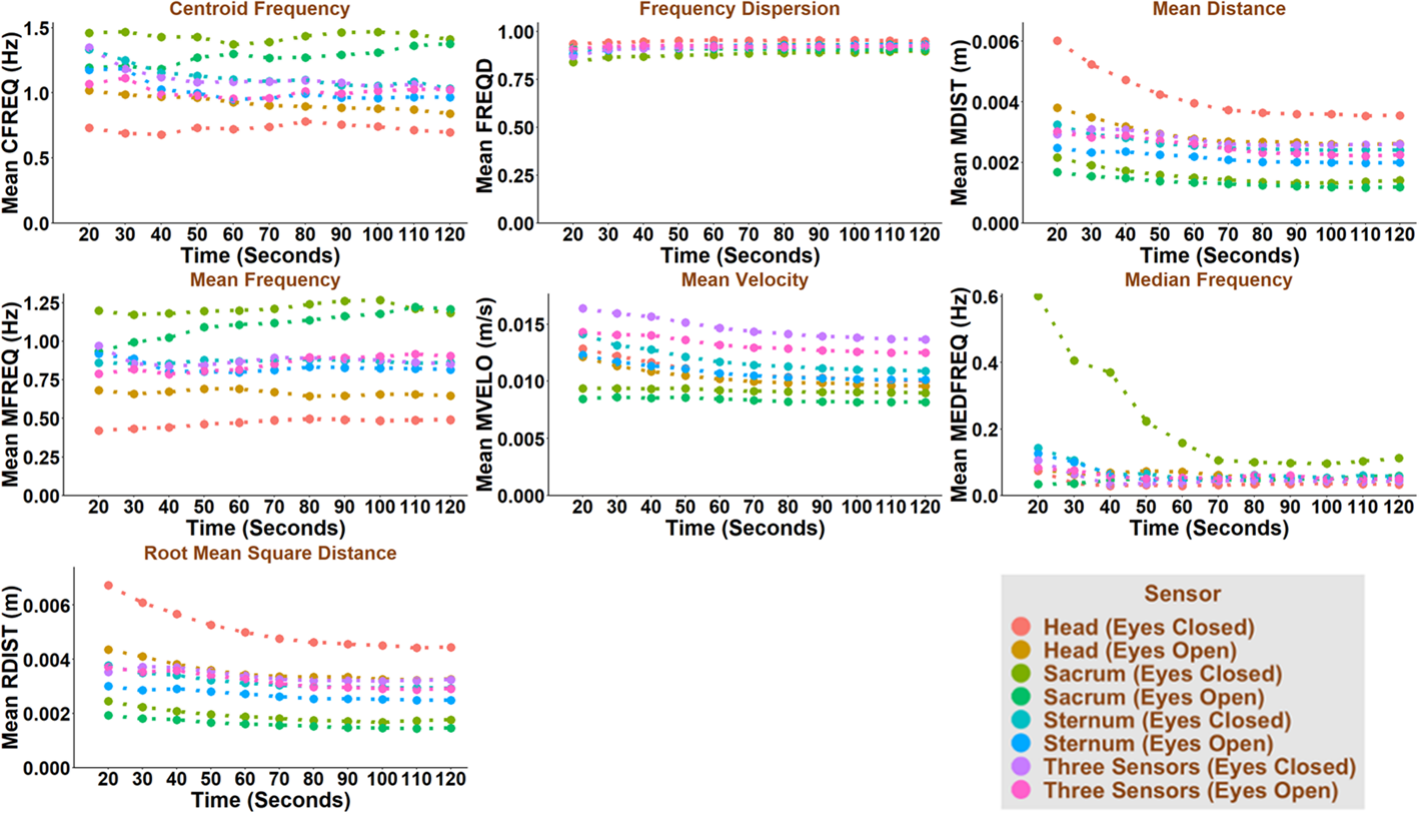
COP-based balance parameters as a function of test duration, test condition and sensor location for mediolateral direction

Test duration, test condition (i.e., eyes open or eyes closed), and sensor location affected most COM-based and COP-based balance parameters (Figs. 1, 2, 3, 4). In general, COM-based parameters tended to be less affected by the test duration than COP-based parameters. Test duration, test condition and sensor location affected the COM-based Centroid Frequency (CFREQ), Mean Frequency (MFREQ), and Median Frequency (MEDFREQ) and the COP-based Frequency Dispersion (FREQD) in the anteroposterior and mediolateral directions less than other parameters. The impact of test duration, test condition and sensor location on balance parameters was comparable in the anteroposterior and mediolateral directions.

### Impact of test duration on the reliability of balance parameters

The ICC values for all balance parameters, together with the standard error of measurement (SEM) and 95% confidence interval (95% CI) are reported in Additional file 1: Table S1. A low to zero SEM was observed for all COM-based and COP-based balance parameters. The reported ICC values for test duration *X* indicate the ICC between the same balance parameter obtained based on the first *X* second of the data (excluding the first 5 s of data at the beginning of the trial for transition effects) and the entire data collected for 120 s. As such, these ICC values quantify the reliability of balance parameters measured in a test duration *X*. When ICC ≥ 0.75 or ICC ≥ 0.90, the balance parameter can be reliably measured in a test duration of *X* for research or clinical purposes, respectively.

Many (but not all) COM-based balance parameters could be reliably measured for research purposes in only 20 s. This means that the ICC between the parameters obtained based on the entire collected data and those obtained based on the first 20 s or longer time windows was always equal to or larger than 0.75. The rest of the COM-based balance parameters (except for FREQD in mediolateral direction and with eyes open condition measured by the sacrum sensor) could be reliably measured for research purposes in 60 s (Table 1). All COP-based balance parameters (except for CFREQ in anterioposterior direction and with eyes-closed condition measured by the head sensor) measured by a sensor on the head or sternum or a three-sensor combination could be reliably measured for research purposes in 70 s. However, none of the COP-based parameters measured with eyes closed using a sensor on the sacrum could be reliably measured in a test duration of even 120 s.

**Table 1:** Minimum test duration (seconds) required to derive reliable COM-based and COP-based balance parameters for research purposes (ICC ≥ 0.75) for different sensor locations and test conditions

Parameter	Eyes	COM				COP			
		Head	Sacrum	Sternum	Three sensor	Head	Sacrum	Sternum	Three sensor
JERK	Open	≥ 50	≥ 20	≥ 20	≥ 20	–	–	–	–
	Closed	≥ 40	≥ 20	≥ 40	≥ 20	–	–	–	–
RMS-ACC	Open	≥ 40	≥ 20	≥ 20	≥ 20	–	–	–	–
	Closed	≥ 20	≥ 20	≥ 40	≥ 20	–	–	–	–
Area-CE	Open	≥ 20	≥ 40	≥ 20	≥ 20	≥ 30	≥ 30	≥ 30	≥ 20
	Closed	≥ 20	≥ 20	≥ 20	≥ 20	≥ 70	≥ 120	≥ 30	≥ 30
Area-SW	Open	≥ 20	≥ 20	≥ 20	≥ 20	≥ 30	≥ 20	≥ 20	≥ 40
	Closed	≥ 20	≥ 20	≥ 40	≥ 20	≥ 30	≥ 120	≥ 40	≥ 20
Anteroposterior direction									
RDIST	Open	≥ 40	≥ 20	≥ 20	≥ 20	≥ 20	≥ 20	≥ 20	≥ 40
	Closed	≥ 20	≥ 20	≥ 20	≥ 20	≥ 20	≥ 120	≥ 20	≥ 20
MDIST	Open	≥ 20	≥ 20	≥ 20	≥ 20	≥ 30	≥ 30	≥ 20	≥ 50
	Closed	≥ 20	≥ 20	≥ 20	≥ 20	≥ 20	≥ 120	≥ 30	≥ 20
MVELO	Open	≥ 20	≥ 20	≥ 20	≥ 20	≥ 20	≥ 20	≥ 20	≥ 20
	Closed	≥ 20	≥ 20	≥ 20	≥ 20	≥ 30	≥ 120	≥ 20	≥ 20
MFREQ	Open	≥ 20	≥ 20	≥ 20	≥ 20	≥ 20	≥ 20	≥ 20	≥ 40
	Closed	≥ 20	≥ 20	≥ 20	≥ 20	≥ 20	≥ 120	≥ 20	≥ 20
MEDFREQ	Open	≥ 20	≥ 20	≥ 40	≥ 60	≥ 20	≥ 40	≥ 20	≥ 20
	Closed	≥ 20	≥ 20	≥ 40	≥ 20	≥ 20	≥ 120	≥ 20	≥ 30
CFREQ	Open	≥ 20	≥ 20	≥ 20	≥ 40	≥ 30	≥ 60	≥ 40	≥ 40
	Closed	≥ 20	≥ 20	≥ 40	≥ 20	≥ 120	≥ 120	≥ 30	≥ 20
FREQD	Open	≥ 40	≥ 20	≥ 40	≥ 40	≥ 20	≥ 30	≥ 30	≥ 50
	Closed	≥ 40	≥ 40	≥ 60	≥ 50	≥ 20	≥ 120	≥ 30	≥ 20
Mediolateral direction									
RDIST	Open	≥ 20	≥ 20	≥ 20	≥ 40	≥ 20	≥ 20	≥ 20	≥ 70
	Closed	≥ 20	≥ 20	≥ 40	≥ 20	≥ 30	≥ 120	≥ 20	≥ 30
MDIST	Open	≥ 20	≥ 20	≥ 20	≥ 40	≥ 30	≥ 20	≥ 30	≥ 60
	Closed	≥ 20	≥ 20	≥ 40	≥ 20	≥ 40	≥ 120	≥ 20	≥ 40
MVELO	Open	≥ 20	≥ 20	≥ 20	≥ 20	≥ 20	≥ 20	≥ 20	≥ 30
	Closed	≥ 20	≥ 20	≥ 40	≥ 20	≥ 20	≥ 120	≥ 30	≥ 30
MFREQ	Open	≥ 20	≥ 20	≥ 20	≥ 20	≥ 20	≥ 30	≥ 40	≥ 70
	Closed	≥ 20	≥ 40	≥ 40	≥ 20	≥ 20	≥ 120	≥ 30	≥ 50
MEDFREQ	Open	≥ 20	≥ 20	≥ 20	≥ 20	≥ 20	≥ 30	≥ 60	≥ 30
	Closed	≥ 20	≥ 40	≥ 20	≥ 20	≥ 30	≥ 120	≥ 40	≥ 50
CFREQ	Open	≥ 20	≥ 60	≥ 20	≥ 20	≥ 30	≥ 40	≥ 60	≥ 70
	Closed	≥ 20	≥ 50	≥ 20	≥ 20	≥ 20	≥ 120	≥ 40	≥ 50
FREQD	Open	≥ 20	≥ 80	≥ 20	≥ 40	≥ 20	≥ 20	≥ 30	≥ 40
	Closed	≥ 20	≥ 40	≥ 20	≥ 50	≥ 20	≥ 120	≥ 20	≥ 40

*Area-CE* 95% confidence ellipse area, *Area-SW* sway area, *CF-ACC* centroid frequency-acceleration, *CFREQ* centroid frequency, *FREQD* frequency dispersion, *JERK* sway jerkiness, *MDIST* mean distance, *MEDFREQ* median frequency, *MFREQ* mean frequency, *MVELO* mean velocity, *RDIST* root-mean-square distance, *RMS-ACC* root-mean-square acceleration

Reliable measurement for clinical purposes (ICC ≥ 0.90) required longer test duration for many balance COM-based balance parameters (Table 2). Yet, most of them (except for FREQD) could be reliably measured in 70 s. Most COP-based balance parameters (except for Area-CE with the eyes-closed condition and CFREQ in anterioposterior direction with the eyes-closed condition, both measured by the head sensor) measured by a sensor on the head or sternum or a three-sensor combination could also be reliably measured for clinical purposes in 100 s. Based on Tables 1 and 2, there is no trend in the impact of test conditions (eyes open vs. eyes closed) on test duration required to obtain reliable balance parameters.

**Table 2:** Minimum test duration (seconds) required to derive reliable COM-based and COP-based balance parameters for clinical purposes (ICC ≥ 0.90) for different sensor locations and test conditions

Parameter	Eyes	COM				COP			
		Head	Sacrum	Sternum	Three sensor	Head	Sacrum	Sternum	Three sensor
JERK	Open	≥ 70	≥ 40	≥ 20	≥ 30	–	–	–	–
	Closed	≥ 60	≥ 40	≥ 50	≥ 40	–	–	–	–
RMS-ACC	Open	≥ 60	≥ 40	≥ 40	≥ 20	–	–	–	–
	Closed	≥ 40	≥ 20	≥ 50	≥ 20	–	–	–	–
Area-CE	Open	≥ 40	≥ 50	≥ 20	≥ 50	≥ 30	≥ 30	≥ 30	≥ 30
	Closed	≥ 50	≥ 40	≥ 20	≥ 40	≥ 120	≥ 120	≥ 60	≥ 40
Area-SW	Open	≥ 50	≥ 40	≥ 20	≥ 50	≥ 50	≥ 50	≥ 50	≥ 60
	Closed	≥ 40	≥ 20	≥ 50	≥ 20	≥ 50	≥ 120	≥ 50	≥ 40
Anteroposterior direction									
RDIST	Open	≥ 60	≥ 40	≥ 40	≥ 20	≥ 40	≥ 40	≥ 40	≥ 50
	Closed	≥ 20	≥ 40	≥ 40	≥ 20	≥ 40	≥ 120	≥ 40	≥ 30
MDIST	Open	≥ 60	≥ 40	≥ 20	≥ 20	≥ 50	≥ 60	≥ 50	≥ 70
	Closed	≥ 20	≥ 40	≥ 20	≥ 20	≥ 50	≥ 120	≥ 50	≥ 40
MVELO	Open	≥ 60	≥ 20	≥ 20	≥ 20	≥ 20	≥ 20	≥ 40	≥ 30
	Closed	≥ 20	≥ 40	≥ 20	≥ 20	≥ 40	≥ 120	≥ 20	≥ 20
MFREQ	Open	≥ 40	≥ 20	≥ 40	≥ 20	≥ 20	≥ 20	≥ 50	≥ 50
	Closed	≥ 20	≥ 20	≥ 40	≥ 40	≥ 30	≥ 120	≥ 20	≥ 20
MEDFREQ	Open	≥ 40	≥ 20	≥ 40	≥ 70	≥ 20	≥ 120	≥ 50	≥ 20
	Closed	≥ 20	≥ 20	≥ 50	≥ 40	≥ 90	≥ 120	≥ 30	≥ 50
CFREQ	Open	≥ 50	≥ 20	≥ 40	≥ 70	≥ 70	≥ 120	≥ 60	≥ 50
	Closed	≥ 20	≥ 20	≥ 40	≥ 40	≥ 120	≥ 120	≥ 50	≥ 20
FREQD	Open	≥ 40	≥ 60	≥ 40	≥ 80	≥ 30	≥ 50	≥ 50	≥ 60
	Closed	≥ 70	≥ 50	≥ 120	≥ 60	≥ 40	≥ 120	≥ 50	≥ 30
Mediolateral direction									
RDIST	Open	≥ 40	≥ 40	≥ 40	≥ 50	≥ 50	≥ 30	≥ 40	≥ 100
	Closed	≥ 50	≥ 20	≥ 60	≥ 40	≥ 40	≥ 120	≥ 30	≥ 60
MDIST	Open	≥ 20	≥ 40	≥ 40	≥ 60	≥ 50	≥ 40	≥ 40	≥ 90
	Closed	≥ 40	≥ 20	≥ 50	≥ 40	≥ 50	≥ 120	≥ 40	≥ 60
MVELO	Open	≥ 20	≥ 40	≥ 20	≥ 50	≥ 20	≥ 30	≥ 30	≥ 40
	Closed	≥ 20	≥ 20	≥ 50	≥ 40	≥ 30	≥ 120	≥ 40	≥ 40
MFREQ	Open	≥ 20	≥ 60	≥ 20	≥ 20	≥ 30	≥ 70	≥ 70	≥ 90
	Closed	≥ 20	≥ 60	≥ 70	≥ 40	≥ 30	≥ 120	≥ 40	≥ 70
MEDFREQ	Open	≥ 20	≥ 60	≥ 20	≥ 50	≥ 40	≥ 40	≥ 70	≥ 40
	Closed	≥ 20	≥ 70	≥ 60	≥ 50	≥ 30	≥ 120	≥ 40	≥ 50
CFREQ	Open	≥ 40	≥ 70	≥ 20	≥ 60	≥ 80	≥ 100	≥ 70	≥ 80
	Closed	≥ 20	≥ 70	≥ 70	≥ 50	≥ 40	≥ 120	≥ 40	≥ 90
FREQD	Open	≥ 40	≥ 100	≥ 90	≥ 60	≥ 30	≥ 60	≥ 40	≥ 70
	Closed	≥ 40	≥ 60	≥ 50	≥ 100	≥ 30	≥ 120	≥ 40	≥ 50

*Area-CE* 95% confidence ellipse area, *Area-SW* sway area, *CFREQ* centroid frequency, *FREQD* frequency dispersion, *JERK* sway jerkiness, *MDIST* mean distance, *MEDFREQ* median frequency, *MFREQ* mean frequency, *MVELO* mean velocity, *RDIST* root-mean-square distance, *RMS-ACC* root-mean-square acceleration

### Correlation between parameters of interest obtained by each sensor

Correlations were observed among COM-based and COP-based balance parameters in different sensor locations and test conditions (Additional file 2: Table S2). In general, most strong correlations were observed among COM-based balance parameters, particularly among time-domain COM-based parameters. However, significant correlations were also observed among COP-based parameters and between COM-based and COP-based parameters.

## Discussion

This study investigated the effects of test duration, the number of sensors, and sensor location on standing balance parameters derived using data from a single accelerometer on the head, sternum, or sacrum and a combination of three accelerometers on the leg, sacrum, and sternum. The results indicated that the required test duration for reliable quantification of COM-based and COP-based balance parameters suggested by Prieto et al. [7] and Mancini [8] depends on the sensor location and testing condition. For research purposes, most COM-based balance parameters could be computed reliably using a minimum test duration of 20 s (excluding 5 s at the beginning and end of each trial) in all testing conditions. Almost all of them could be reliably measured for research purposes (ICC ≥ 0.75) in 60 s (Table 1). Yet, a test duration of 70 s is recommended for clinical purposes (ICC ≥ 0.90), except for FREQD, which results were not reliable that did not obtained reliably results even in a 120-s test duration.

Most COP-based balance parameters could be computed reliably for research purposes (ICC ≥ 0.75) using a minimum test duration of 70 s, when a sensor on the head or sternum or a three-sensor combination was used, except for a few parameters listed in Table 1. The sacrum sensor could not compute most COP-based balance parameters in eyes-closed condition reliably even with a test duration of 120 s. For measurement of most COP-based balance parameters for clinical purposes, a test duration of 100 s should be recommended, except for the sacrum sensor’s rests that were often not reliable (ICC ≥ 0.90) even in a 120-s test duration (Table 2).

The frequency-domain parameters (e.g., CFREQ, MFREQ, MEDFREQ, and FREQD) obtained based on COM and, to some extent, COP were less affected by the test duration than the time-domain parameters (Figs. 1, 2, 3, 4). This might be interpreted as, unlike COM and COP amplitude, their frequency content does not change much throughout the test.

Symptoms that accompany many neurological conditions may fluctuate over time. Therefore, continuous balance assessment is essential for more comprehensive and accurate assessments of the disease’s impact on people’s mobility and ambulatory performance. Although force-plates, computerized dynamic posturography systems, and optoelectronic cameras offer high accuracy and sensitivity to capture balance deficits, their deployment in clinical or community settings is impractical. Furthermore, they do not enable continuous balance assessments. Therefore, an ideal balance assessment tool is one that can be deployed easily and quickly while providing adequate, reliable, and accurate outcomes [37]. Our results indicated that the accuracy of an IMU mounted on head or sternum in balance assessment is comparable with that of a three-IMU combination and a force plate system [12]. Wearable IMUs are thus suitable for continuous, longitudinal assessments of balance [16, 17] and clinical decision-making during neurorehabilitation [18, 21, 38].

Our finding of many but not all COP-based balance parameters for head, sternum and three-sensor combination was consistent with Scoppa et al.’s recommendations for force plate-based measurements that chose the test duration based on the convergence of the COP-based parameter towards a stable value [39] (see Tables 1 and 2 for comparison). They concluded that COP-based balance parameters derived from 25 to 40 s force plate data are steady and reliable [39]. This observation is consistent with the previous research demonstrating that the three-sensor combination provides valid and sensitive metrics of postural sway parameters that correlate well with force-plates [12].

For most COP-based parameters derived from the sacrum sensor when the eyes were closed, a steady trend during the 120-s data collection was not observed. The COP is calculated as a function of the COM acceleration measured by the accelerometer readouts and has a high-frequency content resulting in a higher fluctuation of COP-based parameters, compared to COM-based parameters. The sacrum sensor, particularly, is close to the body COM and thus might provide less predictable information about the COP motion. The lower ICC values in various test durations obtained by the sacrum sensor can also be attributed to the difference between the sway of the sacrum compared to the sternum and head. Therefore, a longer test duration may be needed to use these sensors to collect COP-based parameters reliably. However, longer test duration may add more noise, likely due to fatigue or diminished attention [39]. One solution could be wearing the sensor on the sternum or employing the three-sensor combination. Another solution could be calculating COP-based balance parameters as the average of those obtained in three or more successive recordings. This solution can likely eliminate random temporary effects due to irregular responses [23]. However, it does not eliminate the fatigue effect. Another solution could be selecting one biomarker from each main domain (i.e., one COP biomarker from each of the time-domain distance measures, area measures, time-domain hybrid measures, frequency-domain measures, and acceleration-based measures) as recommended by Prieto [7]. For example, instead of reporting all COP time-domain distance measures, where root-mean-square distance (RDIST) calculation requires a longer data collection, only mean velocity (MVELO) could be reported (Table 2). We also observed high correlations among balance parameters, indicating that some balance parameters carry the same information (Additional file 2: Table S2). Thus, one can select balance parameters with shorter required test duration for research and clinical evaluation without compromising the accuracy of the measurement.

To our knowledge, this is the first study that examined the effect of the sensor location, the number of sensors, and test condition (eyes open vs. eyes closed) on the required test duration for balance parameters measurement using IMUs. Hansen et al. investigated the day-to-day reliability of five different static balance testing conditions in a neuro-geriatric population using an IMU mounted on the lower back [34]. Each test took 30 s and was repeated within a 12- to 24-h interval [34]. Only one balance parameter, acceleration in the mediolateral direction for the semi-tandem stance on a soft surface with eyes open, met the minimum reliability threshold (ICC ≥ 0.70) [34] adequate for research applications [36]. None of the balance parameters met the minimum reliability threshold (ICC ≥ 0.90) adequate for clinical applications [36].

In the present study, we did not directly examine the accuracy of the measurements made by different sensor locations compared to the three-sensor combination. Nevertheless, our cross-correlation and reliability analysis suggest that the outputs of the sensors mounted on the sacrum and sternum are closer to those of the three-sensor combination. Previous studies have indicated that the three-sensor combination provides valid and sensitive metrics of postural sway parameters that correlate well with those obtained by force-plates [12]. Therefore, this combination of three accelerometers could be used as a substitute for force plate systems, particularly for real-time monitoring of an individual’s balance. However, employing only one sensor is more user-friendly in the clinical setting and requires less computational power. This makes them an attractive solution in clinics and remote monitoring of people’s balance.

The recommended test durations in this study are valid based on the COP- and COM-based balance parameters obtained using accelerators. Our previous study [12] showed the body kinematics and COP excursions obtained by these accelerometers were similar to those obtained by motion-capture cameras and a force plate, respectively. Yet, the validity of our recommended test durations must be further studied for balance parameters measured by the cameras and force plate because the repeatability of the balance parameters would depend on the sensor’s noises as well.

We acknowledge a few limitations of the present study. The primary limitation of this study was the relatively small sample size. Despite its small sample size, we did have enough power to detect an ICC of 0.90 or larger, which has been suggested as adequate for use in research conducted using group averages. Also, given the primary purpose of this study, we only recruited volunteers without any prior history of neurological or musculoskeletal diseases or other balance disorders. Notably, neurological and musculoskeletal diseases do not affect every individual in the same way, depending on the stage of the condition and any medications or other care the patient receives. Therefore, employing people with neurological and musculoskeletal diseases would reduce the generalizability of the study outcomes. This would be contrary to the primary objective of the present study, which was to provide recommendations for the minimum required test duration based on sensor location. This limitation, however, warrants further studies that include populations with various clinical conditions.

## Conclusions

The results of the study support our hypothesis that the selection of the minimum test duration for both clinical and research purposes should be made based on the sensor location, number of sensors, and test condition, as well as the specific COM-based or COP-based parameters of interest. To compute COM-based balance parameters using wearables for clinical applications (i.e., with ICC ≥ 0.90), a sensor on the head, sternum or sacrum requires a test duration of no more that 70 s, irrespective of the test condition. Likewise, for computing most of COP-based balance parameters using wearables for clinical applications, a sensor on the head or sternum requires a test duration of no more than 100 s, irrespective of the test condition. A sensor on the sacrum may require a much longer test duration for this latter purpose.

## Methods

### Participants

Twelve volunteers without any prior history of balance disorders (5 females and 7 males) participated in this study. The mean age and weight of the participants were 25.3 ± 4.8 years and 66.1 ± 15.7 kg. The Research Ethics Board of the University of Alberta approved the study in accordance with the Declaration of Helsinki. All participants provided written informed consent before participation.

### Instruments

**Fig. 5:**
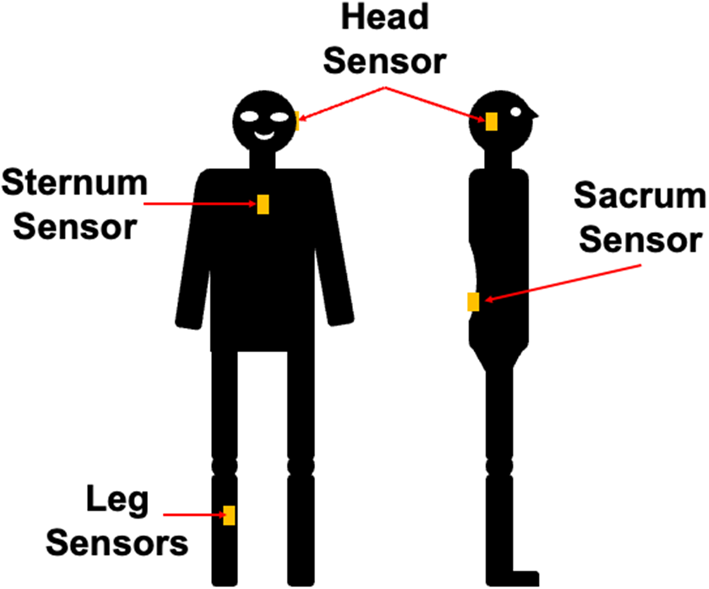
Sensor placement. Inertial measurement units (IMUs) were mounted on the tibia, sacrum, sternum, and head

We used five IMUs (MTws, XSENS Technologies, NL). The IMUs were mounted on the participants’ right tibia, the second sacral vertebra, sternum, and the left mastoid process, one IMU on each location (Fig. 5) [15]. The sampling rate of all sensors was set at 100 Hz. Although all IMUs are equipped with gyroscopes, accelerometers, and magnetometers, we only used accelerometer data in this study. Our previously validated algorithm can estimate COM and COP information from a single IMU or a combination of readouts from two or more IMUs. Therefore, we estimated COM and COP information for a single sensor placed on the sacral vertebra, sternum, and the left mastoid process, as well as a combination of three sensors placed on the right tibia, the second sacral vertebra, and sternum [12].

### Procedure

Participants were instructed to stand still with their feet shoulder-width apart and hands at their sides during testing. Each participant was tested for 2 min with their eyes open and again for 2 min with their eyes closed. We chose a 2-min duration because it was one of the longest test durations used in the literature [8] and yet was not reported to cause fatigue or loss of attention.

### Data and statistical analysis

Data post-processing was carried out offline using an algorithm previously developed by our team [12, 21, 38], implemented in MATLAB (MathWorks, USA). The algorithm estimates time- and frequency-domain balance parameters using COP and COM time series obtained by only one accelerometer or a combination of three accelerometers [12, 21, 38].

We used single- and multi-segment inverted pendulum models to estimate the body’s COM velocity [12]. A single-segment inverted pendulum model was employed to estimate COM-based and COP-based parameters for data derived from a single sensor (i.e., head, sternum, or sacrum). Likewise, a multi-segment inverted pendulum model was deployed for data derived from the three-sensor combination. In brief, using the accelerometer data, we obtained the instantaneous orientation of the body segments above the ankle joint [12]. The COM position was then calculated based on the segments’ orientation and their length [12]. Finally, we estimated the COM mean velocity by dividing the total COM excursion into the time duration, according to Prieto et al. [7]. This method eliminates drift problems that arise when the velocity is calculated via time integration of the accelerometer readout.

Estimating the COP-based balance parameters involved several steps. In summary, first, the anthropometric parameters of each body segment, such as the mass, COM, joint centers of rotation, and moments of inertia, were estimated using the individual’s body mass and height according to Winter’s method [6]. Second, the accelerometer readout was employed to estimate the instantaneous inclination angle of each body segment above the ankle joint in the single- or multi-segment inverted pendulum model [12]. Third, a top-down inverse dynamic approach was deployed to estimate joint moments and forces on the ankle joint, assuming that the only external force acting on the body was the ground reaction force (GRF) [12]. Fourth, Newton–Euler’s equations of motion for the foot were used to estimate GRF and COP position [12].

Balance parameters of interest were calculated for the single head, sternum, and sacrum accelerometers and the three-sensor combination (right leg, sacrum, and sternum) for both COP and COM acceleration. These parameters include time-domain distance measures (i.e., root-mean-square distance [RDIST], mean distance [MDIST], and mean velocity [MVELO]), area measure (i.e., 95% confidence ellipse area [Area-CE]), time-domain hybrid measures (i.e., sway area [Area-SW] and mean frequency [MFREQ]), frequency-domain measures (i.e., median frequency [MEDFREQ], centroid frequency [CFREQ], and frequency dispersion [FREQD]), and COM’s acceleration-based measures (i.e., sway jerkiness [JERK], and root-mean-square acceleration [RMS-ACC] [7, 8]. We revised the original definition of JERK presented by Mancini et al. [8] and normalized the Jerk value by the test duration to eliminate the effect of recording time.

These sensor locations were chosen because they were used in the literature. The sacrum sensor was chosen as it is the closest point to the body COM [8] and may provide the most accurate estimation of the COM movements [8]. The sternum sensor was chosen because several studies have used it to monitor daily living activities [15, 16, 40, 41]. Thus, placing the sensor on the sternum may allow monitoring balance and activities of daily living using just one sensor. We chose the head sensor because recent studies have shown that head sensors can enable sensitive measurements of balance impairments and sensory reweighting due to head injuries [35, 42]. We employed the three-sensor combination, to use a four-segment model of the body incorporating the ankle, hip and low back joints. This model provides valid and sensitive metrics of postural sway parameters, given the significant role of these joints in maintaining standing balance [12]. As such, we included a sensor on the tibia (and not on the thigh) together with sensors on the sacrum and sternum to calculate the COM and COP trajectories using this four-segment model [12].

To examine the effect of test duration, the accelerometer data were analyzed and processed to obtain balance parameters recommended by Prieto [7] and Mancini [8] using COP and body COM acceleration for different test duration (20, 30, 40, 50, 60, 70, 80, 90, 100, 110, and 120 s) using the data obtained during the same 120-s trial. For a 20-s duration, after removing the initial 5 s from 120-s data, the first 20 s of the data were extracted. The same procedure was repeated for the rest of the test durations (i.e., 30, 40, 50, 60, 70, 80, 90, 100, and 110 s). We chose these segments from the beginning of the 120-s data to consistently study the start of standing trial and prevent the impact of fatigue or loss of attention.

The measurement reliability for each test duration was assessed using the intra-class correlation coefficients (ICC) to compare the parameter computed for each test duration with that obtained using data for the full 120-s test duration. The present study aimed to compare the reliability of shorter 20-, 30-, 40-, 50-, 60-, 70-, 80-, 90-, 100-, and 110-s test durations with the full 120-s data. A high ICC value indicates a strong agreement (correlation) between the segments, suggesting that the information carried by the shorter segments is consistent with and similar to the complete data. While comparing the parameters obtained by the complete data to those obtained by shorter versions of the data will naturally produce high levels of correlation, the purpose of using ICC in this context is not to establish a comparison with external reference data, but rather to evaluate the internal consistency of the data. This assessment provides important new insights into the impact of test duration on the reliability and stability of standing balance measurements using wearable sensors.

The sensor location was considered the fixed effect. The participants were considered the random effect. Portney has suggested that ICC values between 0.75 and 0.90 indicate good reliability and are suitable for research purposes [36]. In contrast, ICC values greater than 0.90 indicate excellent reliability, suitable for clinical purposes [36]. The 95% confidence interval for all balance parameters of interest derived from COM and COP information was calculated. Similarly, the standard error of measurement (SEM) was calculated to estimate measurement error in the measurement unit $({\text{SEM}}={\text{SD}}\sqrt{1-{\text{ICC}}})$, where SD represents the standard deviation of the measurement [43]. A low SEM indicates that the variability or margin of error associated with these balance parameters is minimal, suggesting that the data collected can be considered highly accurate and consistent [43].

A post hoc Pearson’s cross-correlation analysis was conducted among all balance parameters to determine those that provide the same information when dealing with postural balance. The level of significance was set at 0.05. All statistical analyses were conducted using R software, version 3.6 [44].

### Supplementary Information

**Additional file 1: Table S1.** Reliability of the COM-based and COP-based balance parameters obtained in test duration of 20, 30, 40, 50, 60, 70, 80, 90, 100, and 110 s, expressed as the intra-class correlation (ICC) between these parameters and those obtained based on the complete data (120 s). The ICC values, 95% confidence interval (95% CI) and standard error of measure (SEM) are reported for both test conditions (i.e., eye open or eyes closed) and for all sensor locations (i.e., head, sacrum, sternum, and three-sensor combination).

**Additional file 2: Table S2.** Correlation among COM-based balance parameters, among COM-based balance parameters, and between COM-based and COP-based balance parameters in both test conditions (i.e., eye open or eyes closed) and for all sensor locations (i.e., head, sacrum, sternum, and three-sensor combination).

## Data Availability

Data could be made available upon request through a collaborative process. Please contact the corresponding author for further details.

## Abbreviations

Area-CE: 95% Confidence ellipse area
Area-SW: Sway area
CFREQ: Centroid frequency
COM: Center of mass
COP: Center of pressure
FREQD: Frequency dispersion
ICC: Intraclass correlation coefficient
JERK: Sway jerkiness
MDIST: Mean distance
MEDFREQ: Median frequency
MFREQ: Mean frequency
MVELO: Mean velocity
RDIST: Root-mean-square distance
RMS-ACC: Root-mean-square acceleration
